# Transcriptomics-proteomics Integration reveals alternative polyadenylation driving inflammation-related protein translation in patients with diabetic nephropathy

**DOI:** 10.1186/s12967-023-03934-w

**Published:** 2023-02-06

**Authors:** Tingting Zhao, Dongdong Zhan, Shuang Qu, Song Jiang, Wenhua Gan, Weisong Qin, Chunxia Zheng, Fang Cheng, Yinghui Lu, Mingwei Liu, Jinsong Shi, Hongwei Liang, Yi Wang, Jun Qin, Ke Zen, Zhihong Liu

**Affiliations:** 1 National Clinical Research Center of Kidney Diseases, Jinling Hospital, Nanjing University School of Medicine, Nanjing, 210002 Jiangsu China; 2 State Key Laboratory of Proteomics, Beijing Proteome Research Center, National Center for Protein Sciences (Beijing), Beijing Institute of Lifeomics, Beijing, 102206 China; 3 State Key Laboratory of Pharmaceutical Biotechnology, Nanjing University School of Life Sciences, Nanjing, 210093 Jiangsu China

**Keywords:** Diabetic nephropathy, Glomeruli, Alternative polyadenylation, Transcriptomics, Proteomics

## Abstract

**Background:**

Diabetic nephropathy (DN) is a complex disease involving the upregulation of many inflammation-related proteins. Alternative polyadenylation (APA), a crucial post-transcriptional regulatory mechanism, has been proven to play vital roles in many inflammatory diseases. However, it is largely unknown whether and how APA exerts function in DN.

**Methods:**

We performed transcriptomics and proteomics analysis of glomeruli samples isolated from 50 biopsy-proven DN patients and 25 control subjects. DaPars and QAPA algorithms were adopted to identify APA events from RNA-seq data. The qRT-PCR analysis was conducted to verify 3′UTR length alteration. Short and long 3ʹUTRs isoforms were also overexpressed in podocytes under hyperglycemia condition for examining protein expression.

**Results:**

We detected transcriptome-wide 3′UTR APA events in DN, and found that APA-mediated 3ʹUTR lengthening of genes (APA genes) increased their expression at protein but not mRNA level. Increased protein level of 3′UTR lengthening gene was validated in podocytes under hyperglycemia condition. Pathway enrichment analysis showed that APA genes were enriched in inflammation-related biological processes including endoplasmic reticulum stress pathways, NF-κB signaling and autophagy. Further bioinformatics analysis demonstrated that 3′UTR APA of genes probably altered the binding sites for RNA-binding proteins, thus enhancing protein translation.

**Conclusion:**

This study revealed for the first time that 3′UTR lengthening of APA genes contributed to the progression of DN by elevating the translation of corresponding proteins, providing new insight and a rich resource for investigating DN mechanisms.

**Supplementary Information:**

The online version contains supplementary material available at 10.1186/s12967-023-03934-w.

## Introduction

Diabetic nephropathy (DN), the leading cause of end-stage renal disease (ESRD), is a complicated inflammatory disease characterized by multiple layers of regulation [1–3]. Many inflammation-related proteins, enriched in endoplasmic reticulum stress pathways, NF-κB signaling, autophagy, cell-cell adhesion and vesicle-mediated transport, are upregulated and promote the development and progression of DN [4–6]. Numerous investigations exploring the pathogenesis of DN have been carried out from the perspective of genetic alterations, dysregulated pathways and metabolic dysfunctions [7–11]. However, it remains unclear how these inflammation-associated proteins are upregulated at the post-transcriptional level in DN.

Alternative polyadenylation (APA), a crucial post-transcriptional regulatory mechanism, has been reported to occur in most human genes [12, 13]. By selecting different polyadenylation sites (polyA sites, PAS) in 3’UTR, 3’UTR-APA generates distinct transcripts with variable 3’UTR lengths [12, 13]. Many cis-regulatory elements, such as microRNA (miRNA) or RNA-binding protein (RBP) binding sites, are embedded in the 3ʹUTR sequence, therefore, the presence or absence of these cis-acting elements conferred by 3ʹUTR-APA has far-reaching effects on the stability, translation rate, nuclear export, cellular localization of target mRNAs, and cellular localization of proteins [12]. APA-associated genetic variants have been proposed to affect diverse physiological and pathological processes [14]. For instance, the global shortening of 3′UTRs has been reported in many proliferative diseases, such as oculopharyngeal muscular dystrophy, immune disorders and various cancers [15–19]. The genes regulated by APA tend to use proximal PAS in 3′UTRs, and APA-mediated 3′UTR shortening may allow these genes to escape the inhibitory effects of miRNA by losing their corresponding binding sites [17]. On the contrary, global lengthening of 3′UTRs has been detected in cell differentiation and development processes, including embryonic development and differentiation of myoblasts, embryonic stem cells and neurons [20–22]. Therefore, the corresponding RBPs can bind to specific linear sequence motifs or secondary structures located within the lengthened 3′UTR to modulate the subcellular localization and translation efficiency of mRNAs [23–26]. However, up to date, there is little information available about APA-mediated 3′UTR shortening or lengthening in the progression of DN.

The goal of this study was to characterize the existence of 3ʹUTR-APA and its potential role in the development and progression of DN. To achieve this, we performed an integrated analysis of transcriptomics and proteomics of glomeruli isolated from 50 biopsy-proven DN patients and 25 control subjects. Two different algorithms, DaPars and QAPA, were adopted to construct the landscape of human 3′UTR APA events using RNA-seq datasets. We found that DN glomeruli exhibited genome-wide APA and nearly 95% of APA-regulated genes used distal poly(A) sites in 3′UTRs compared to that in control glomeruli. Those genes with 3′UTRs lengthening were mainly enriched in inflammation-related biological processes. Further integration of transcriptomics and proteomics profiling, combined with experimental validation, revealed that APA-mediated 3ʹUTR lengthening in DN glomeruli increased the protein levels but not mRNA levels of the target genes. Collectively, our study revealed for the first time the APA-induced 3′UTR lengthening of inflammation-associated genes in glomeruli under DN condition and a critical role of APA-induced 3′UTR lengthening in elevating the protein translation of corresponding genes.

## Research design and methods

### Human samples and biospecimens collection

Fifty biopsy-proven DN patients enrolled in this study were from the Renal Biobank of National Clinical Research Center of Kidney Diseases, Jiangsu Biobank of Clinical Resources. All DN patients were diagnosed by renal biopsy without other diseases. The baseline clinical characteristics were collected within 1 month of renal biopsy (Additional file 1: Table S1). Among them, 31 (64%) patients were males and the other 19 (36%) were females. The median age was 47.5 (range, 33–67 years). The eGFR (estimate glomerular filtration rate) was calculated using the CKD-EPI (Chronic Kidney Disease Epidemiology Collaboration) formula [27], and the median eGFR was 70.04 (range, 23.5–114.1 ml/min1.73m2). The median 24 h urine protein was 2.35 (range, 0.29–13.32 g/24 h). The median SBP (systolic blood pressure) was 136 (range, 114–157 mmHg) and DBP (diastolic blood pressure) was 80 (range, 69–98 mmHg), respectively. The median Scr (serum creatinine) was 1.16 (range, 0.54–2.86 mg/ dL) and BUN (blood urea nitrogen) was 20.65 (range, 8.8–50.4 mmol/L). As it is essentially inaccessible of normal kidney biopsy samples, we enrolled 25 age- and gender-matched nondiabetic renal cell carcinoma patients as controls. The control subjects were absence of proteinuria and displayed eGFR > 90 mL/min and normal levels of serum creatinine and blood urea nitrogen. In detail, 16 (64%) patients were males and 9 (36%) patients were females. The median age was 47 (range, 25–67 years) and the median eGFR was 125.6 (range, 92.9–137.3 ml/min1.73m2). The median Scr was 0.69 (range, 0.39–1.08 mg/ dL) and BUN was 5.27 (range, 3.48–6.87 mmol/L). The median SBP and DBP were 123 (range, 109–150 mmHg) and 77 (range, 68–100 mmHg), respectively. The DN kidney samples were obtained from the leftover portions of routine renal biopsy biopsies and control tissues were obtained from tumor-free tissues which were greater than 5 cm away from the surgical margin. The pathological sections were examined by three professional pathologists independently, and they draw the consistent conclusion that the pathological characteristics of each control sample conform to normal (Additional file 2: Fig. S1). The biopsies were stored at − 80 °C until use. We performed high-throughput RNA sequencing (RNA-Seq) and proteomics LC-MS/MS analysis for each DN patient and control subject. For each biopsy sample, 40–50 glomeruli were manually microdissected under a stereomicroscope, and 20 glomeruli were randomly selected for transcriptomics and proteomics analysis, respectively. This study was approved by the Institutional Review Board of Jinling Hospital (Nanjing, China). All participants signed informed consent form of using clinical specimens for medical research.

### RNA-seq analysis

The high-throughput RNA sequencing (RNA-Seq) of 20 glomeruli from each biopsy sample was performed using the Illumina comprehensive next-generation sequencing (NGS) technique. The quality control and data filtering were carried out by the FastQC software (Version 0.11.5). After removing the low-quality RNA-Seq reads (Phred quality score less than 20 or nucleotides less than 50), the filtered reads were mapped onto the human reference genome (GRCh38.p12. genome, released on 12/2013) using HISAT2 software (Version 2.1.0) [28]. Assembly and quantification of the transcripts were conducted based on a reference human genome annotation file with StringTie software (Version 1.3.1) [29]. Fragments Per Kilobase of transcript per Million mapped read (FPKM) was used for the measurements of the relative quantification of the transcripts. The maximal FPKM values  ≥ 1 were defined as an effectively expressed transcripts.

### Protein extraction, trypsin digestion and LC-MS/MS analysis

For the proteomics detection, 20 glomeruli from each biopsy sample were lysed in a buffer containing 1% deoxycholic acid sodium salt, 40 mM CAA, 10mM Tris Hydrochloride and pH8.5, protease and phosphatase inhibitors (Thermo Scientific), followed by 5 min of sonication (3s on and 3s off, amplitude 25%). The lysate was centrifuged at 15,000×g for 10 min at 4 °C, and the supernatant was collected as whole tissue extract (WTE). Protein concentration was determined by Nanodrop protein assay. WTE was digested with trypsin [30]. Digested peptides were then injected into the column and eluted using a gradient of 5–35% acetonitrile, for 150 min. The resulting peptides were analyzed on a Q Exactive HF Hybrid Quadrupole-Orbitrap Mass Spectrometer. The MS/MS analysis was performed under a data-dependent mode. One full scan was followed by up to 20 data-dependent MS/MS scans with higher-energy collision dissociation (normalized collision energy of 35%) or collision-induced dissociation (normalized collision energy of 27%). Dynamic exclusion time was set at 18 s.

### Peptide identification and protein quantification

Raw sequencing data were searched against the National Center for Biotechnology Information (NCBI) Ref-seq human proteome database in Firmiana implemented with the Mascot search engine (Matrix Science, version 2.3.01) [31]. The mass tolerances were set as 20 ppm for precursor ions and 0.05 Da for product ions, N-acetylation and oxidation of methionine were set as variable modifications, and cysteine carbamidomethylation was set as a fixed modification. The peptide FDR was 1%. Proteins with at least one unique peptide and two strict peptides or more than two strict peptides (mascot ion score ≥ 20) were selected for further analysis. A label-free, intensity-based absolute quantification (iBAQ) approach was used to calculate protein quantification. For each sample, protein iBAQ values were further normalized to fraction-of-total (FOT) [32]. The FOT was further multiplied by 10^5^ to obtain iFOT for easy presentation of low abundant proteins. To further increase the reliability, we selected the proteins detected in at least one-tenth of the samples for subsequent analysis.

### DaPars algorithm to predict APA events

We adopted DaPars algorithm to predict APA events from conventional RNA-seq data. DaPars performed *de novo* identification and quantification of dynamic APA events between DN and controls, regardless of any prior APA annotation [33]. Percentage of Distal polyA site Usage Index (PDUI) was defined as the ratio of the expression level of transcripts with distal PAS to the sum of the transcripts with distal PAS and proximal PAS. The greater the PDUI was, the more distal polyA site of a transcript was used. The median PDUI difference between DN and control (ΔPDUI, DN-control) was calculated to detect dynamic APAs events, which was capable of identifying lengthening (distal PAS in DN, ΔPDUI > 0.1, P-value  ≤ 0.05) or shortening (proximal PAS in DN, ΔPDUI < − 0.1, P-value ≤ 0.05) 3ʹUTRs.

### QAPA algorithm to predict APA events

Quantification of APA (QAPA) algorithm, a faster and more sensitive new approach to quantitatively infer APA from conventional RNA-seq data based on a greatly expanded resource of poly(A) site annotations [34], was adopted to verify APA events predicted by DaPars. Distal poly(A) site usage (DPAU) was defined as the relative expression of distal 3′UTR isoforms over the total expression of all detected 3′UTR isoforms. The median change in DPAU (ΔDPAU, DN-control) was calculated between DN and control. Genes with ΔDPAU > 10 and P-value ≤ 0.05 were deemed to have lengthening 3′UTRs, while ΔDPAU < **− **10 and P-value ≤ 0.05 were considered as 3′UTRs shortening.

### Cell culture and plasmid transfection

Immortalized human podocytes (HPCs) provided by Dr. Moin Saleem (University of Bristol, Bristol, UK) were cultured as previously described [35]. The cells were initially cultured in RPMI-1640 medium supplemented with 10% FBS and Insulin-Transferrin-Selenium (ITS) (Gibco) at 33 °C for proliferation. The CDS region of CYB5R1 were PCR-amplified from HPC cDNA using I-5 High-Fidelity Master Mix (Tsingke, China) and the primers listed in Additional file 3: Table S2, and then inserted between the BamH1 and short or long 3’UTRs sites. The short and long 3’UTRs of CYB5R1 were PCR-amplified from HPC genomic DNA using I-5 High-Fidelity Master Mix (Tsingke) and the primers listed in Additional file 3: Table S2, and inserted into the construct between the CYB5R1 CDS region and NotI site. To overexpress the long and short 3′UTR isoforms of CYB5R1, 10^5^ cells were transfected with 2 µg pcDNA3.1(+)-CYB5R1-SUTR and pcDNA3.1(+)-CYB5R1-LUTR plasmid using lipofectamine^™^ 3000 (Invitrogen) for 6 h, respectively. The stable cell lines were generated through multiple rounds of selection against puromycin treatment. Cells were then switched to 37 °C for 10–14 days to induce differentiation. Finally, the differentiated podocytes were treated with high concentration of glucose (HG) (60 mmol/L) for 24 h.

### Western blot analysis

Total proteins were extracted from cells using RIPA buffer containing protease inhibitor cocktail (Roche). The protein lysates were separated by SDS-PAGE and transferred to polyvinylidene fluoride membranes. After blocked with 5% nonfat milk, the membranes were incubated with the primary antibody of CYB5R1 (Proteintech, #11807-1-AP). Finally, the membrane was incubated with the horseradish peroxidase–labelled secondary antibody, followed by color development using ECL Plus detection system (Vazyme, USA).

### RT-PCR analysis

Total RNA was extracted from 20 glomeruli of each biopsy sample (Six control and six DN) using RNAeasy mini kit (Cat#74,004, QIAGEN Science, Germantown, MD) according to the manufacturer’s instruction. The cDNA was synthesized with the first-strand cDNA synthesis kit (Amersham, Buckmgahamshire, UK) and PCR was performed using the Geneamp PCR system (Sigma). The primers for the two isoforms of each gene with different 3′UTR lengths (CYB5R1-L, CYB5R1-S and PDLIM1-L, PDLIM1-S) were listed in Additional file 3: Table S2. The short primer was common to total APA isoforms (S + L) and the long primer was specific to the longer isoform (L). The relative expression of the two isoforms was quantified by qRT-PCR with GAPDH as internal reference.

### Bioinformatics analysis and statistical analysis

Both analyses were performed with R language version 3.40. HC (Hierarchical clustering), PCA (Principal component analysis) and t-SNE (t-distributed stochastic neighbor embedding) analysis were used to visualize the separation between DN and control. Shapiro-Wilk test was adopted to check for fit with a normal distribution, and differential expression analysis was conducted by the Wilcoxon test in combined with FDR adjustment. Fold change (FC, DN/C) ≥ 2 or ≤ -2 and FDR ≤ 0.01 were defined as significant differential expressions. The protein-per-mRNA FC ratio was calculated as the ratio of protein FC to mRNA FC for each gene to evaluate protein translation [36]. Gene Ontology (GO) term enrichment analysis was conducted based on the Database for Annotation Visualization and Integrated Discovery (DAVID) version 6.8 and Fisher’s exact test [37]. Correlation analyses were calculated by Spearman’s correlation coefficients (*r*). The data for western blot and RT-PCR were presented as the mean ± SEM (standard error of mean).

## Results

### The APA landscape in glomeruli from biopsy-proven DN patients

**Fig. 1:**
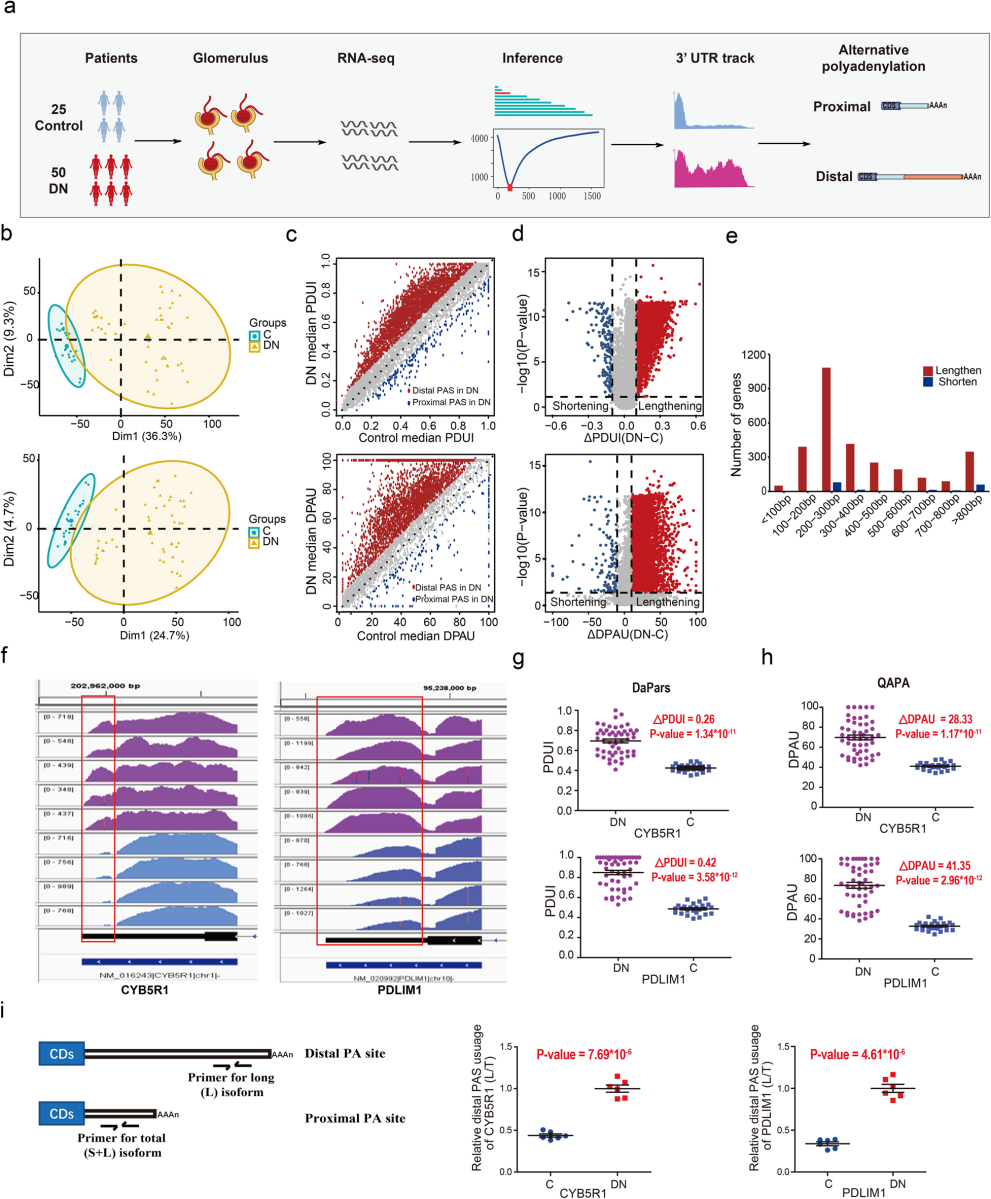
Global lengthening of 3′UTRs in DN identified from RNA-seq data and qRT-PCR verification. **a** The workflow for the identification of dynamic APA events from glomerular RNA-seq data. **b** PCA analysis of PDUI (top) and DPAU (bottom) score clearly separated DN patients from control subjects. **c** Scatter plots of median PDUI (top) or DPAU (bottom) scores between DN and control for each gene. Dashed lines represent ± 0.1 cutoffs for PDUI and ± 10 cutoffs for DPAU, respectively. The different 3′UTR isoforms resulted from the usage of distal PAS (red) or proximal PAS (blue) in DN were colored. **d** The volcano plots of 3ʹUTR lengthening (red) and shortening (blue) genes. The cut off value was ∆PDUI (DN-C) = ± 0.1 and -log10 (P-value) = 1.301 (1.301 corresponds to P-value = 0.05) for DaPars algorithm (top), ∆DPAU (DN-C) = ± 10 and -log10 (P-value) = 1.301 for QAPA algorithm (bottom). **e** The histogram showed the number of genes with 3ʹUTR lengthening and shortening due to APA. **f** Two representative RNA-seq tracks of dynamic APA-regulated genes (CYB5R1, PDLIM1) to highlight the 3′UTR coverage differences between DN and control samples. Purple track represented DN and blue track represented control. The red box indicated the different part of distal 3ʹUTR. **g**–**h**, The quantification of PDUI (g) and DPAU (h) changes for CYB5R1 (top) and PDLIM1 (bottom) between DN and control samples. **i** qRT-PCR analysis to verify the changes of distal and proximal PASs usage of CYB5R1 and PDLIM1 between DN and control. Schematic diagrams of the primer pair design were illustrated in left panel. The increased usage of distal PASs (L/T) in DN compared with control were quantified (middle and right panel). The data represented the mean ± SEM of six independent experiments

To construct the dynamic APA landscape in glomeruli between DN patients and control subjects, we utilized two different algorithms, DaPars [33] and QAPA [34], to identify APA events directly using RNA-seq datasets of glomeruli isolated from 50 biopsy-proven DN patients and 25 controls (Fig. 1a). PDUI and DPAU values were respectively calculated using DaPars and QAPA methods to measure the proportion of distal PAS usage for each gene in DN and control samples (Additional file 4: Table S3.1 and S3.2; Additional file 2: Fig. S2a). PCA analysis based on PDUI (Fig. 1b, top) and DPAU (Fig. 1b, bottom) values showed that the significant difference in 3′UTR lengths clearly distinguished DN glomeruli from the control, indicating the existence of dynamic APA events in DN and control glomeruli. Then, the ΔPDUI and ΔDPAU scores between DN and control were obtained to detect DN-associated 3′UTR length alterations (Additional file 4: Table S3.1 and S3.2). As shown in Fig. 1c and d (top), DaPars revealed 2835 genes with 3′UTR lengthening (ΔPDUI > 0.1, P-value < 0.05) and 169 genes with 3′UTR shortening (ΔPDUI < − 0.1, P-value < 0.05). The finding that DN glomeruli had significantly more 3′UTR lengthened genes (95%, 2,835/3,004) than shortened ones (5%, 169/3,004) was further confirmed by QAPA analysis, which revealed that 3,340 and 129 genes were lengthened (ΔDPAU > 10, P-value ≤ 0.05) and shortened (ΔDPAU < − 10, P-value ≤ 0.05) in 3′UTR, respectively (Fig. 1c, d, bottom). The DN-associated lengthening events were predominantly within the length of 200–300 bp fragment sequences (Fig. 1e).

The visualization of RNA-seq tracks of the representative dynamic APA-regulated genes, such as CYB5R1, PDLIM1, PDCD6, MYOF and CFH, confirmed the longer 3′UTRs tracks mainly in DN glomeruli but not in control. Compared with DN samples, the distal 3′UTR tracks in control renal samples almost disappeared, but the proximal 3′UTR tracks remained unchanged (Fig. 1f; Additional file 2: Fig. S2b). These differential 3′UTRs tracks between DN and control were consistent with the ΔPDUI and ΔDPAU scores calculated by DaPars and QAPA algorithms (Fig. 1g–h; Additional file 2: Fig. S2c, d), respectively. To verify the alteration of 3′UTR length underlying DN, qRT-PCR analysis was conducted using CYB5R1 and PDLIM1 genes, with the primers for the two isoforms being designed based on the 3’UTR sequence differences (Fig. 1i, left). The short primer was common to both APA isoforms (shorter and longer, S + L) and the long primer was unique to the longer isoform (L). The qRT-PCR results clearly showed a greater usage percentage of the distal PAS for CYB5R1 and PDLIM1 genes in DN glomeruli compared to that of the control ones (Fig. 1i, middle and right). Collectively, these results suggested that DN glomeruli exhibited a genome-wide APA and nearly 95% of APA-regulated genes used distal poly(A) sites in 3′UTRs. Further GO enrichment results demonstrated that those genes with 3′UTRs lengthening were mainly enriched in inflammation-related biological processes such as endoplasmic reticulum stress pathways, NF-κB signaling, autophagy, cell-cell adhesion and vesicle-mediated transport, etc. The representative 3′UTR lengthened genes enriched in these biological processes were listed in Table 1.

**Table 1:** The significant biological processes enriched by 3′UTR lengthened genes in DN

Biological process	Count	FDR		Representative Genes
cell-cell adhesion	125	2.92E-09		TWF1, PDLIM1, MYO6, MPRIP, TMOD3, CALD1
macroautophagy	112	2.77E-05		PRKAA1, TOMM22, LAMTOR1, MTDH, DCN
endoplasmic reticulum stress	42	5.45E-05		THBS1, PDIA4, TMX1, TMEM33, UFC1, UFM1
positive regulation of NF-κB signaling	66	5.85E-03		SLC44A2, TMED4, CTNNB1, MYD88, BST2, RELA
ER to Golgi vesicle-mediated transport	78	1.34E-06		LMAN1, PROS1, CUL3, ERGIC1, CTSC, NSF, VAPA
intracellular protein transport	103	3.51E-06		PDCD6, SNX2, ARFIP1, AP3M1, AP2S1, CLTB
vesicle-mediated transport	73	5.46E-06		CLTB, CLINT1, STX12, PLIN3, RAB14, AP3S1
mRNA splicing, via spliceosome	93	1.04E-04		NUDT21, PRPF8, SNRPB2, ELAVL1, DHX15, PPIL3
protein folding	78	2.11E-04		TXNL1, PFDN2, PPIL3, GRPEL1, LTBP4, ERP29
protein stabilization	62	4.57E-04		SUMO1, WFS1, ATP1B3, STX12, PDCD10, BUB3
ubiquitin-dependent protein catabolic	153	2.26E-05		PSMD2, PSME3, PCNP, PCBP2, BUB3, STT3B

### The existence of post-transcriptional regulatory mechanisms in DN glomeruli

**Fig. 2:**
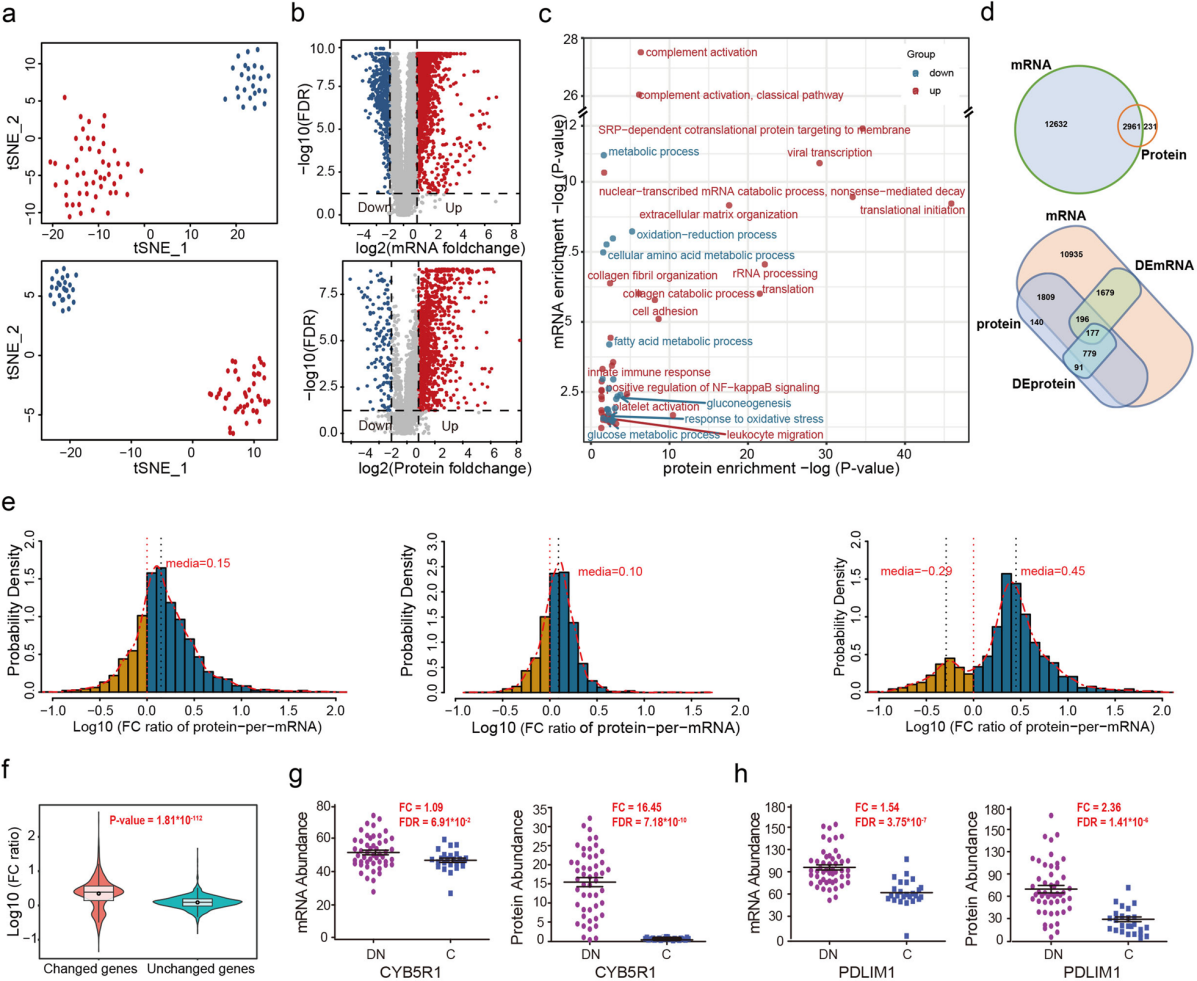
Higher protein translation in DN glomeruli compared to controls. **a** The t-SNE analyses of transcriptomic (top) and proteomic (bottom). Red nodes represented DN patients and blue nodes represented controls. **b** The volcano plot of transcriptomic (top) and proteomic (bottom). The differentially expressed genes (2-fold, FDR ≤ 0.01) were highlighted with red (upregulated) and blue (downregulated). **c** Two-dimensional annotation of biological process (BP) enrichment analysis of differential proteins and mRNAs. The signed -log10 (P-value) values of the BP enrichment at the protein and mRNA level are indicated in the x and y axes, respectively. Red nodes represented the BPs enriched by upregulated mRNAs and proteins, whereas blue nodes represented the BPs enriched by downregulated mRNAs and proteins. **d** Venn diagram showed the number of matched mRNA-protein pairs (top) as well as the overlap of significantly differentially expressed (DE) mRNA and protein (bottom). **e** Protein-per-mRNA FC ratio analysis illustrated the fold-change discordance between mRNA and the corresponding protein. The value of FC ratio was log10 transformed. The FC ratio value for 2961 matched genes (left) and 1809 unchanged genes (middle) were log-normally distributed; The FC ratio analysis for 1152 differentially expressed genes identified two populations of extreme values (right). **f** The violin plot showed the difference in protein-per-mRNA FC ratio between the differentially expressed genes and the non-differentially expressed genes. P-value was calculated by Mann-Whitney U test. **g–h** The mRNA (left) and protein (right) scatterplots for CYBR1 (g) and PDLIM1 (h) between DN and control samples-h The mRNA (left) and protein (right) scatterplots for CYBR1 (g) and PDLIM1 (h) between DN and control samples

Given that APA is a critical part of post-transcriptional regulation, we next determined the post-transcriptional regulation in DN. First, we obtained unbiased glomerular proteomics profiles in parallel with RNA-seq-based transcriptomics to compare the landscape differences between proteomics and transcriptomics. A total of 15,593 mRNAs and 3192 proteins with high confidence were identified, respectively (Additional file 5: Table S4.1 and S4.2). Both t-SNE (t-distributed Stochastic Neighbor Embedding) and unsupervised HC (Hierarchical Clustering) analysis demonstrated a clear separation between DN patients and controls both in transcriptomics (Fig. 2a, top; Additional file 2: Fig. S3a) and proteomics (Fig. 2a, bottom; Additional file 2: Fig. S3c). Further differential expression analysis identified 2,052 mRNAs (FDR ≤ 0.01, fold change > 2), among which 1,547 mRNAs were upregulated and 505 ones were downregulated in DN (Fig. 2b, top; Additional file 2: Fig. S3b; Additional file 5: Table S4.3). Meanwhile, a total of 1047 significantly differentially expressed proteins were identified, with 931 upregulated and 116 downregulated in DN (Fig. 2b, bottom; Additional file 2: Fig. S3d; Additional file 5: Table S4.4). Many well-known DN-associated genes, such as TFGB1, HSPG2, FN1, COL14A1, COL6A2, C3, SYNPO, NPHS1 and NPHS2 [4, 5] displayed significant differential expression between DN and control glomeruli in both mRNA and protein levels, indicating the reliability of the datasets. (Additional file 2: Fig. S3e and S3f). Further GO enrichment analysis demonstrated that both the upregulated mRNAs and proteins were significantly enriched in complement activation, immune response, inflammation, NF-κB signaling, collagen catabolic process, cell adhesion, extracellular matrix organization, translation, mRNA catabolic process, and leukocyte migration etc. The downregulated mRNAs and proteins were clustered in various metabolic processes involving glucose, fatty acids and amino acids, as well as in some oxidation-reduction processes (Fig. 2c).

Subsequently, we integrated the transcriptomics and proteomics profiles to systematically explore expression differences between proteins and their corresponding mRNAs. A total of 2,961 matched mRNA-protein pairs were selected in our cohort (Fig. 2d, top). The Spearman’s correlation coefficients of expression levels and expression changes (DN/control) for those mRNA-protein pairs were both relatively weak (Additional file 2: Fig. S3g and Fig. S3h). Among the 2961 matched mRNA-protein pairs, 177 genes were differentially expressed at mRNA and protein levels, while 196 mRNAs and 779 proteins were differentially expressed at mRNA or protein levels (Fig. 2d, bottom; Additional file 2: Fig. S3i). To compare the FC (fold change, DN/C) discordance between mRNA and their corresponding protein, the protein-per-mRNA FC ratio for each gene was calculated. The analysis results demonstrated that, for 2961 mRNA-protein matched genes, the median value of FC ratio was 0.15 (Fig. 2e, left; Additional file 6: Table S5.1). Among them, the median FC ratio of 1809 unchanged genes was 0.10 (Fig. 2e, middle; Additional file 6: Table S5.2). However, for 1152 differentially expressed genes, their FC ratio showed a bimodal pattern, with median values of 0.45 and − 0.29, respectively (Fig. 2e, right; Additional file 6: Table S5.3), and the proportion of genes with positive FC ratio was significantly greater than that with negative FC ratio. In addition, there was also a significant FC ratio discrepancy (P-value = 1.80 × 10^− 112^, Mann-Whitney U-test) between the differentially expressed genes and the unchanged ones (Fig. 2f). Interestingly, the DN-associated genes CYB5R1, PLIM1, PDCD6, MYOF and CFH, all possessing a lengthened 3′UTR in DN glomeruli, exhibited significantly increased protein levels but unchanged mRNA levels compared to that in control samples (Fig. 2g–h; Additional file 2: Fig. S4).

### Contribution of APA-induced 3′UTR lengthening to the increase of protein translation of DN-associated genes

**Fig. 3:**
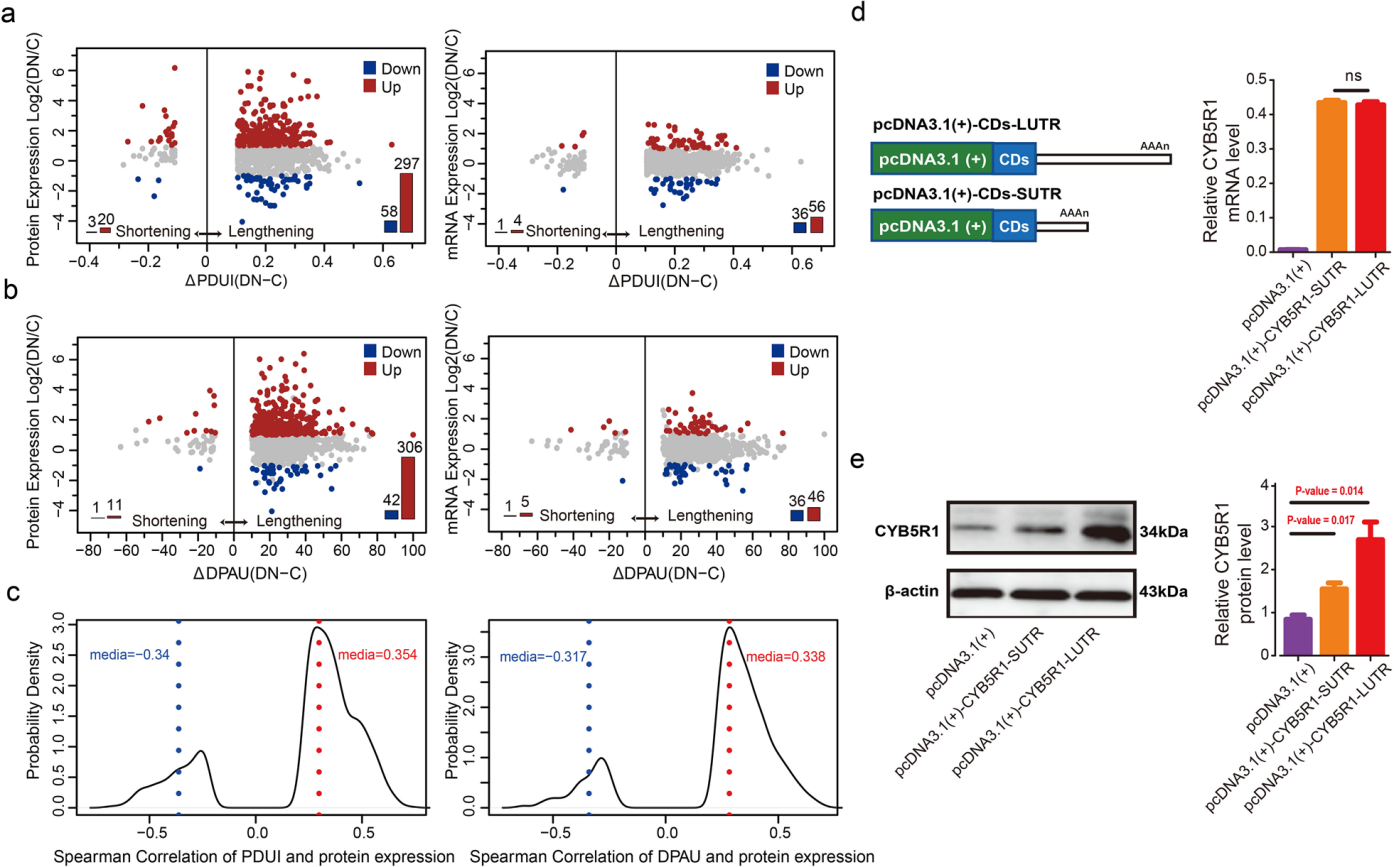
The APA-induced 3′UTR lengthening promoted protein translation. **a** Scatterplots between ΔPDUI (DN-C) and expression changes in proteins (left) and mRNAs (right) levels for the mRNA-protein matched genes with significantly longer (ΔPDUI > 0.1, P-value ≤ 0.05) and shorter 3′UTRs (ΔPDUI < − 0.1, P-value ≤ 0.05). The genes were significantly upregulated (red) or downregulated (blue) (2-fold) in DN, respectively. **b** Scatterplots between ΔDPAU (DN-C) and expression changes in proteins (left) and mRNAs (right) levels for the mRNA-protein matched genes with significantly longer (ΔDPAU > 10, P-value ≤ 0.05) and shorter 3′UTRs (ΔDPAU < − 10, P-value ≤ 0.05). **c** The density plots of the statistically significant spearman correlation coefficient (P-value < 0.05) between PDUI (left) or DPAU (right) score and protein abundance for the mRNA-protein matched genes. The dashed lines represented the median value of positive (red) and negative (blue) correlation coefficient. **d**, **e** The experimental validation of the APA-mediated 3ʹUTR lengthening in DN increasing protein translation. Schematic diagrams of the pcDNA3.1(+)-CYB5R1-LUTR and pcDNA3.1(+)-CYB5R1-SUTR plasmid constructs were illustrated in d (left panel). The qRT-PCR (d, right panel) and western blot (e) of CYB5R1 in human podocytes under hyperglycemia were performed after transfecting cells with a plasmid expressing long or short 3′UTR isoform. The data represented the mean ± SEM of three independent experiments.

Given that the cis-acting elements in 3′UTRs sequence control crucial post-transcriptional regulation processes [26, 38, 39], we then investigated whether the APA-induced global lengthening of 3′UTRs in DN glomeruli would affect protein translation. The combination of APA results and omics data indicated that a higher number of 3ʹUTR lengthened genes were upregulated at proteins levels compared with mRNA levels (Fig. 3). DaPars revealed that 31.4% (297/947) of the 3ʹUTR lengthened genes increased at protein levels, but just 5.9% (56/947) of them increased at mRNA levels (Fig. 3a, Additional file 2: Fig. S5a). Similarly, the QAPA algorithm demonstrated that 30.8% (306/993) of the 3ʹUTR lengthened genes were upregulated at protein levels, whereas 4.6% (46/993) of them were upregulated at mRNA levels (Fig. 3b; Additional file 2: Fig. S5b). It was suggested that APA-mediated 3ʹUTR lengthening was associated with the increase of protein translation in DN. Furthermore, more genes showed a significant positive correlation between the proportion of distal PAS usage and their corresponding protein abundance, which may be attributed to the fact that the longer 3ʹUTR contains more additional regulatory elements that can enhance protein translation (Fig. 3c).

Next, to experimentally verify the influence of APA events on protein translation, we overexpressed different isoforms of CYB5R1 mRNA with short or long 3ʹUTRs in human podocytes stimulated with high concentration of glucose (HG), and then detected the differences in mRNA and protein expression. As depicted in Fig. 3d (left), we cloned the CYB5R1 coding DNA sequence (CDS) into pcDNA3.1(+) plasmid and fused it with either long or short 3′UTR. Podocytes transfected with various CYB5R1-expressing plasmids were then exposed to high glucose. The qRT-PCR analysis of CYB5R1 after plasmid transfection indicated that the mRNA levels of CYB5R1 isoforms with short or long 3ʹUTRs were similar (Fig. 3d, right). However, western blot analysis showed that the long 3ʹUTR isoform expressed significantly more CYB5R1 protein than the short isoform and control pcDNA3.1(+) plasmid under hyperglycemic conditions (Fig. 3e). Briefly, these results corroborated the conclusion that APA-mediated 3ʹUTR lengthening can contribute to enhancing protein translation in DN.

### Potential regulatory mechanisms of poly(A) site selection and translation enhancement in DN

**Fig. 4:**
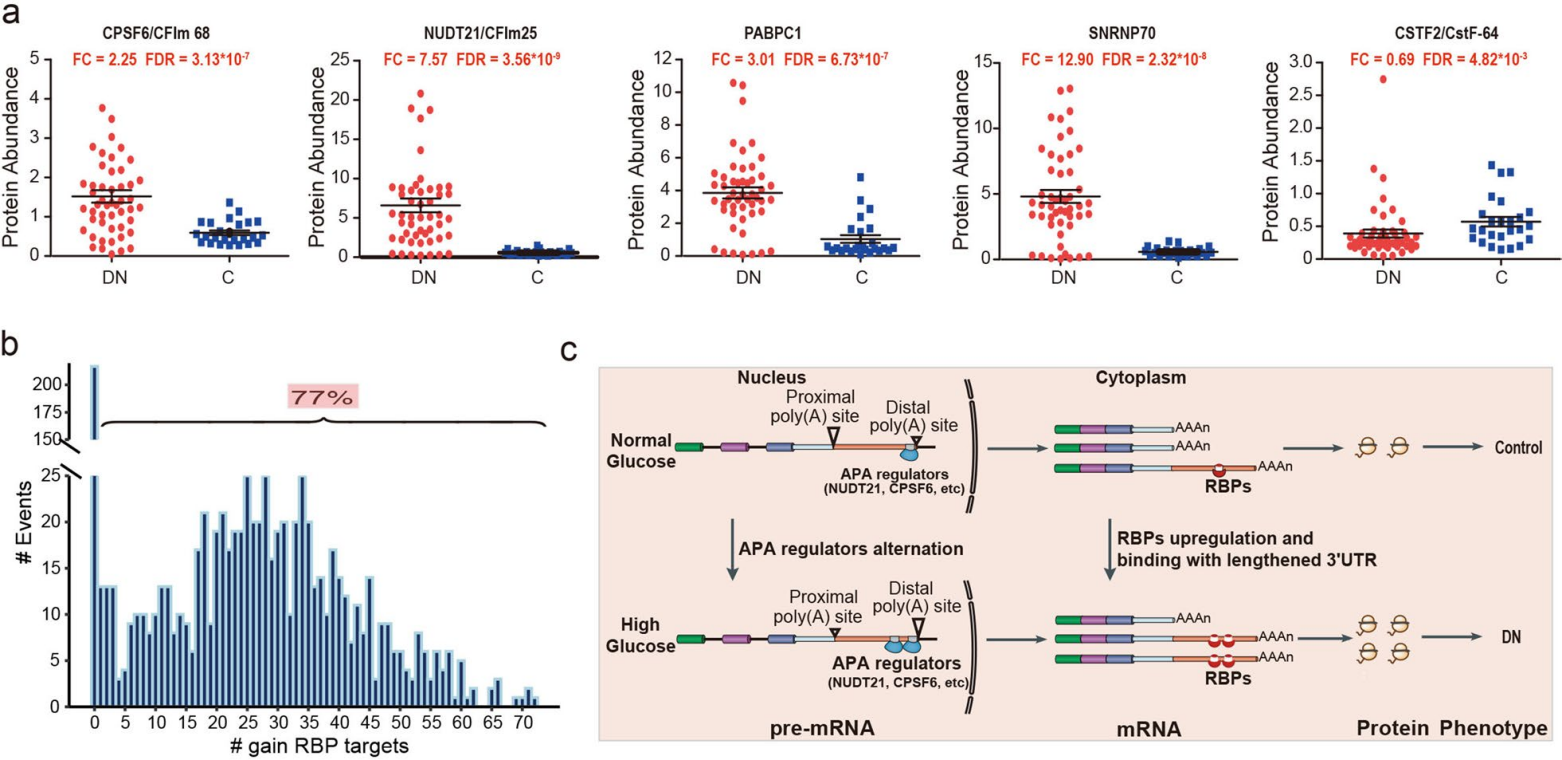
The potential regulation mechanisms of PAS selection and translation enhancement in DN. **a** The protein scatterplots of the representative polyadenylation factors (NUDT21, CPSF6, SNRNP70, PABPC1 and CstF64) between DN patients and control subjects. **b** The number of genes gaining RBP-binding sites due to the lengthening of 3′UTR. 77% 3′UTR lengthened genes have gained at least one predicted RBP binding site. **c** The schematic of 3′UTR lengthening increasing gene translation in DN. Genes such as CYB5R1 and PDLIM1 preferred the use of proximal PAS under normal conditions. In DN, upregulated polyadenylation factors (NUDT21, CPSF6, SNRNP70, and PABPC1) resulted in higher usage of distal PASs and thus increased the abundance of the isoform with longer 3′UTR, which produced more protein through gaining more RBP binding sites

A growing number of core polyadenylation factors have recently been identified as regulators in PAS selection [13, 33, 40]. To determine the APA regulators behind DN, we observed the protein expression changes of 22 important APA regulators according to our proteomic data (Additional file 2: Fig. S5c). Among these APA-regulatory factors, several factors promoting the selection of distal PAS were expressed at higher levels in DN compared to control subjects (Additional file 2: Fig. S5c). For example, CFIm complex (Cleavage Factor Im complex), a heterodimer consisting of CFI25/CPSF5/NUDT21 and CFI68/CPSF6 or CFI59/CPSF7, were reported to preferentially bind to distal PAS, and the upregulation of CFIm subunits promoted distal PAS usage [41, 42]. Other factors, such as SNRNP70 (as a component of the multi-subunit RNP U1 snRNP) and polyadenylate-binding protein 1 (PABPC1), exhibited similar effects on increasing distal PAS site usage, and upregulation of these factors led to polyadenylation at distal sites [43–45]. In contrast, CstF64/CSTF2 has an opposite function, with CstF64 reduction enhancing the usage of distal PAS sites [16, 33]. As shown in Fig. 4a, we found that the abundance of CFIm factors, SNRNP70 and PABPC1 at the protein level was strongly enhanced in DN patients, whereas these proteins were nearly absent in control subjects. However, the protein level of CstF64 was lower in DN, although the difference was not significant between DN patients and controls (Fig. 4a). These results suggested that CFIm factors, SNRNP70 and PABPC1, but not CstF64, served as potential master regulators of distal PAS usage in DN. These APA regulators may be the potential therapeutic targets for DN patients.

By choosing distal PASs in DN, APA-mediated 3′UTR lengthening could provide more binding sites for miRNA or RBPs. Due to the binding of miRNA exerted translation repressive effect, we speculated that RBPs, but not miRNAs, were the master regulators in DN. Meanwhile, previous studies have demonstrated that protein translation could be regulated by RBPs [46, 47]. Therefore, to explore whether the increased protein translation of APA-induced 3′UTR lengthening genes in DN was due to the enhanced interactions with RBPs, we first calculated the median difference of FC ratio (log2△FC ratio) between RBPs-bound and RBPs-unbound genes. The RBP binding information was extracted from the POSTAR2 database, which is the largest post-transcriptional regulation database including RBP-binding sites derived from various CLIP-seq datasets [48]. The results revealed that among 169 RBPs, 26 RBPs were identified to significantly improve protein translation in DN (log2∆FC ratio > 0, P-value < 0.05), including NOP56, FUBP3, FBL, EWSR1, FXR1, TAF15 and HNRNPA1 (Additional file 7: Table S6.1-6.2). According to our proteomic data, 15 RBPs were detected, 14 of which were upregulated or unchanged at the protein level in DN (Additional file 7: Table S6.3). Many of the those RBPs that promote translation in DN are well-known translational regulatory genes, such as NOP56, FBL, and FUBP3, which have been reported to improve protein translation in various biological processes [24, 49, 50]. These results supported the view that RBPs can regulate the protein translation of DN-associated genes by interacting with cis-acting elements in 3′UTR sequence.

Next, we evaluated whether the obtaining of more RBP binding sites from 3′UTR lengthening could enhance protein translation. To determine the global patterns of APA-mediated increases in RBP binding sites, we searched for the gained RBP binding sites for all 3′UTR lengthened genes. The results demonstrated that ~ 80% (726/943) genes with a lengthened 3′UTR in DN gained at least one predicted RBP binding site compared to control 3′UTR, implying that RBPs may play a critical role in increasing the protein translation of DN-associated genes with a lengthened 3′UTR in DN (Fig. 4b). The representative DN-associated genes with improved protein translation, which bound to the selected translational enhancer RBPs through an extended 3′UTR sequence, were listed in Additional file 7: Table S6.3. Consequently, our results suggested that the DN-associated genes with APA-regulated 3′UTR lengthening had higher protein translation, because they possessed more RBP-binding sites and were more likely to be regulated by certain translational enhancer RBPs (Fig. 4c).

## Discussion

As the leading cause of ESRD, DN development and progression is controlled by multiple layers of gene regulation [1–3]. Many inflammation-related proteins are upregulated in the progress of DN, [4–6]; however, the post-transcriptional regulatory mechanism that enhances the protein expression of these DN-associated genes remains unclear. Alternative polyadenylation (APA), a crucial post-transcriptional regulatory mechanism, has been proven to exert vital roles in many inflammatory diseases [15–26]. However, whether and how APA exerts function underlying DN is largely unknown. In this study, we demonstrated the existence of APA-mediated 3ʹUTR lengthening of inflammation-associated genes in DN glomeruli, and highlighted its role in enhancing protein translation.

To avoid bias in APA analysis, we utilized two different algorithms, DaPars and QAPA, to systematically construct the atlas of dynamic APA events, using RNA-seq datasets of glomeruli isolated from 50 biopsy-proven DN patients and 25 control subjects. DaPars is a *de novo* analysis that discovers APA events regardless of any prior APA annotation, whereas QAPA algorithm demarcates 3′UTR sequences that are specifically affected by APA based on a more comprehensive resource of annotated PAS [33, 34]. Our results indicated that two different algorithms reached a consistent conclusion, that was, APA induced 3′UTR global lengthening peculiar to DN patients, which was also verified by qRT-PCR and the visualization of 3′UTR RNA-seq tracks. These APA-regulated 3′UTR lengthening genes were mainly enriched in inflammation-related biological processes such as NF-κB signaling, endoplasmic reticulum stress, vesicle-mediated transport, autophagy, and cell-cell adhesion. To explore the potential molecular mechanisms that control the global lengthening of 3ʹUTRs in DN, we investigated protein alterations of the core polyadenylation factors that regulate PAS selection. The results demonstrated that some APA-regulatory factors that promoted distal PAS usage, including CFIm25, CFIm68, SNRNP70, and PABPC1, were highly expressed in DN glomeruli compared to controls. Such findings were in consistent with previous studies, in which these polyadenylation factors have been reported to bind with the sequences near distal PAS, thereby preventing the usage of proximal PAS [41, 43–45]. Depletion of these APA-regulatory factors markedly increased the selection of proximal PAS and led to 3ʹUTR global shortening [13, 42, 45]. These results provided the evidence that APA was a possible regulatory mechanism underlying 3′UTR lengthening during DN pathogenesis, and CFIm25, CFIm68, SNRNP70 and PABPC1 served as potential master regulators in distal PAS usage.

Based on previous reports that 3ʹUTR-APA could affect the stability and translation of target mRNA as well as the cellular localization of proteins, the discovery of APA-induced 3′UTR global lengthening in DN raises the question as to whether the 3′UTR length alteration could regulate the expression of DN-associated gene. To answer this question, we integrated APA results with proteomics and transcriptomics profiles and found that a greater number of 3ʹUTR lengthened genes were upregulated at the protein level compared to the mRNA level. Specifically, ~ 31.0% of the 3ʹUTR lengthened genes were increased at the protein level, but just ~ 5.0% were increased at the mRNA level. Correlation analyses also demonstrated that the proportion of distal PAS usage could reflect protein abundance in DN. The molecular experiments also strengthened the evidence on this subject. As presented in Fig. 3d–e, we overexpressed different isoforms of CYB5R1 mRNA with short or long 3′UTRs in human podocytes under hyperglycemia condition to compare the mRNA and protein expression differences. After stimulation with high concentration of glucose, compared with the short isoform, the long 3′UTR isoform resulted in a significant increase in protein expression without a significant difference in mRNA level. Therefore, these results corroborated the conclusion that APA-mediated 3ʹUTR lengthening in DN could increase protein translation.

Mechanistically, 3′UTR sequence contain cis-acting regulatory elements that bind miRNAs or RBPs, and the presence or absence of these cis-acting elements through 3ʹUTR-APA could influence gene expression [51]. For instance, by escaping the repressive effect of miRNAs, proto-oncogenes with 3ʹUTR shortening display increased gene expression, leading to the activation of proto-oncogenes in cancer cells [18, 52]. However, the global lengthening of 3′UTRs in cell differentiation and development processes could confer greater potential for RBPs binding and influence subcellular localization and protein translation [23–26]. For example, Gau et al. proved that the RBP of FUBP3 interacted with FGF9 3’UTR to promote FGF9 mRNA translation [24]. Berkovits et al. reported that compared to the short 3’UTRs isoform, the long 3’UTRs isoforms of CD47, CD44, ITGA1 and TNFRSF13C were bound by HuR and transported to cell surface [23]. Accordingly, we speculated that RBPs, but not miRNAs, were the master regulators for the translation enhancement of inflammation-associated genes underlying DN. In support of this hypothesis, the combination of 3′UTR alterations with the RBP binding site database showed that ~ 80% of genes with lengthened 3′UTRs in DN gained at least one predicted RBP binding site compared to the controls, and the protein translation of the genes with increased RBPs binding sites within the lengthened 3′UTR were significantly improved. Therefore, as depicted in Fig. 4c, our results supported the view that the increases in RBP-mediated regulation led to enhanced protein translation of APA-regulated 3′UTR lengthened genes in DN. Interestingly, it is worth noting that the finding of APA-induced 3′UTR lengthening of inflammation-associated genes in DN and its role in enhancing protein expression were inconsistent with the reports of tumor studies, in which enhanced oncogene expression by APA-induced 3′UTR global shortening was observed [17, 33]. These differences may suggest that the development and progression of DN and tumor are governed by different APA regulatory mechanisms. This may be attributed to the fact that, unlike tumor, which is generally considered highly immunosuppressive, DN more associated with chronic inflammation [4, 53].

Our research revealed for the first time the landscape of APA in DN from RNA-seq datasets of glomeruli using two different algorithms. The integration of proteomics profile with the APA landscape, combined with experimental validation, highlighted the role of APA-induced 3′UTR lengthening in enhancing protein translation. Of course, this study has its limitations. Firstly, due to the restriction of proteomics detection techniques, the number of proteins detected in proteomic is much less than that of transcriptomics. Secondly, the number of patients enrolled is relatively small. To obtain clear and unbiased information about the APA landscape in DN, more biopsy samples should be included. Finally, our findings suggest that the alteration of RBP binding is involved in upregulation of certain inflammation-related proteins in DN. The detailed molecular basis underlying such regulation, however, remains unclear and requires further study.

## Conclusion

Our comprehensive integration of the proteomics profile with the APA landscape inferred from RNA-seq and experimental validation revealed for the first time that APA-induced 3′UTRs lengthening of inflammation-associated genes contributed to protein translation by altering RBPs binding. By illustrating the potential mechanisms that govern the upregulation of various inflammation-associated genes in DN pathogenesis, this study provided novel therapeutic targets for DN. Of course, follow-up research should be carried out to screen the APA regulatory molecules identified in the present study, and to expand the clinical cohort for validation the role of APA-induced 3′UTRs lengthening in DN pathogenesis.

## Supplementary Information

**Additional file 1: Tables S1.** The clinic characteristics for DN patients and controls.

**Additional file 2:** Supplementary Figures. **Figure S1.** The pathological images for control samples. **Figure S2.** The global lengthening of 3′UTRs in DN and the representative examples of dynamic APA-regulated genes. **Figure S3.** Transcriptomics and proteomics analysis of glomeruli isolated from DN patients and controls. **Figure S4.** The mRNA and protein expression changes between DN and control for the represent APA-regulated genes. **Figure S5.** The global lengthening of 3′UTRs and the protein expression changes of the core polyadenylation factors in DN.

**Additional file 3: Tables S2.** The primers for qRT-PCR and overexpression vector.

**Additional file 4: Tables S3.** The dynamic APA events calculated by Dapars and QAPA algorithms.

**Additional file 5: Tables S4.** The transcriptomics and proteomics data analysis.

**Additional file 6: Tables S5.** The protein-per-mRNA FC ratio analysis.

**Additional file 7: Tables S6.** The RBPs that significantly improved protein translation underlying DN.

## Data Availability

MS raw files and searching output data are deposited into proteomeXchange with the accession number IPX0003092000; RNA-Seq data are deposited into the NCBI Sequence Read Archive (SRA) with the accession number PRJNA732573. The data that support the findings of this study are available within the paper and its Supplemental files. All other data are available from the corresponding authors on reasonable request.

## Abbreviations

DN: Diabetic nephropathy
APA: Alternative polyadenylation
RBPs: RNA binding proteins
ESRD: End-stage renal disease
3'UTR: 3′untranslated region
PAS: Poly(A) sites
t-SNE: t-distributed stochastic neighbor embedding
HC: Hierarchical clustering
GO: Gene ontology
PCA: Principal component analysis
FC: Fold change
TE: Translation efficiency
HG: High concentration of glucose
PABPC1: Polyadenylate-binding protein 1.
