# Supplementary material for: Transcriptomics-proteomics Integration reveals alternative polyadenylation driving inflammation-related protein translation in patients with diabetic nephropathy
Source: J Transl Med. 2023 Feb 6;21:86. doi: 10.1186/s12967-023-03934-w (PMC9900993; doi:10.1186/s12967-023-03934-w)
Supplement: Supplementary file 2 — Additional file 2: Supplementary Figures. Figure S1. The pathological images for control samples. Figure S2. The global lengthening of 3′UTRs in DN and the representative examples of dynamic APA-regulated genes. Figure S3. Transcriptomics and proteomics analysis of glomeruli isolated from DN patients and controls. Figure S4. The mRNA and protein expression changes between DN and control for the represent APA-regulated genes. Figure S5. The global lengthening of 3′UTRs and the protein expression changes of the core polyadenylation factors in DN. [file 12967_2023_3934_MOESM2_ESM.pdf]

SUPPLEMENTARY MATERIAL FOR

Transcriptomics-proteomics Integration Reveals Alternative Polyadenylation Driving Inflammation-related Protein Expression in Patients with Diabetic Nephropathy

Tingting Zhao[1#], Dongdong Zhan[2#], Shuang Qu[3#], Song Jiang[1], Wenhua Gan[3], Weisong Qin[1], Chunxia Zheng[1], Mingwei Liu[2], Fang Cheng[2], Yinghui Lu[1], Jinsong Shi[1], Hongwei Liang[3], Yi Wang[2], Jun Qin[2]*, Ke Zen[3]*, Zhihong Liu[1]*

Corresponding authors: Zhihong Liu, M.D. (Email: liuzhihong@nju.edu.cn)
Ke Zen, Ph.D. (Email: kzen@nju.edu.cn)
Jun Qin, Ph.D. (Email: jqin1965@126.com)

This PDF file includes:

Supplementary Figures S1 to S5
Legends for Supplementary Table S1 to S6

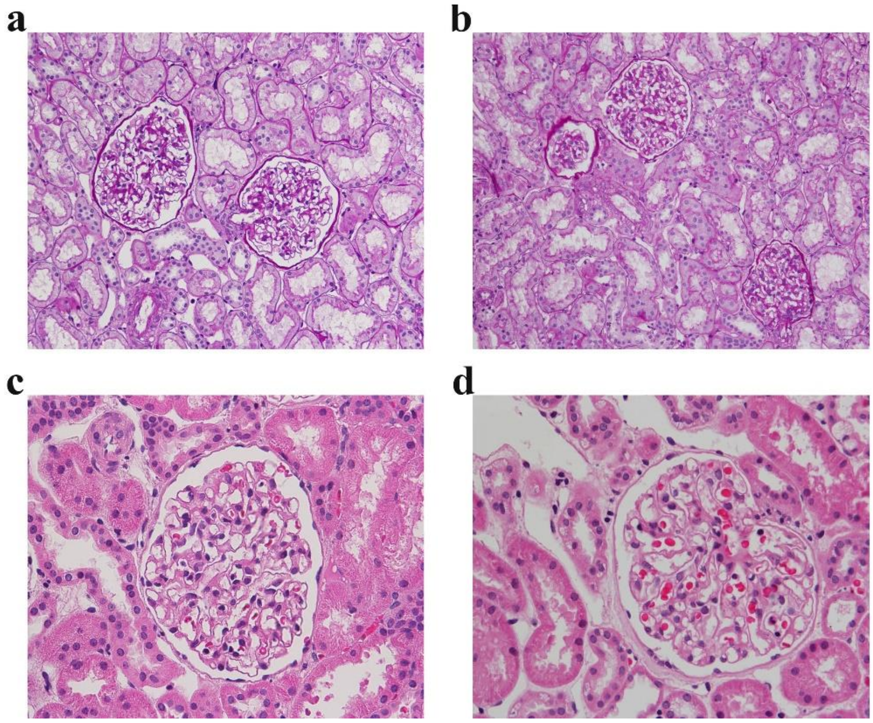

Figure S1. The pathological images for control samples.

The pathological images of the control samples were consistent with the pathological features of normal glomeruli. (×200 for a and b, ×400 for c and d)

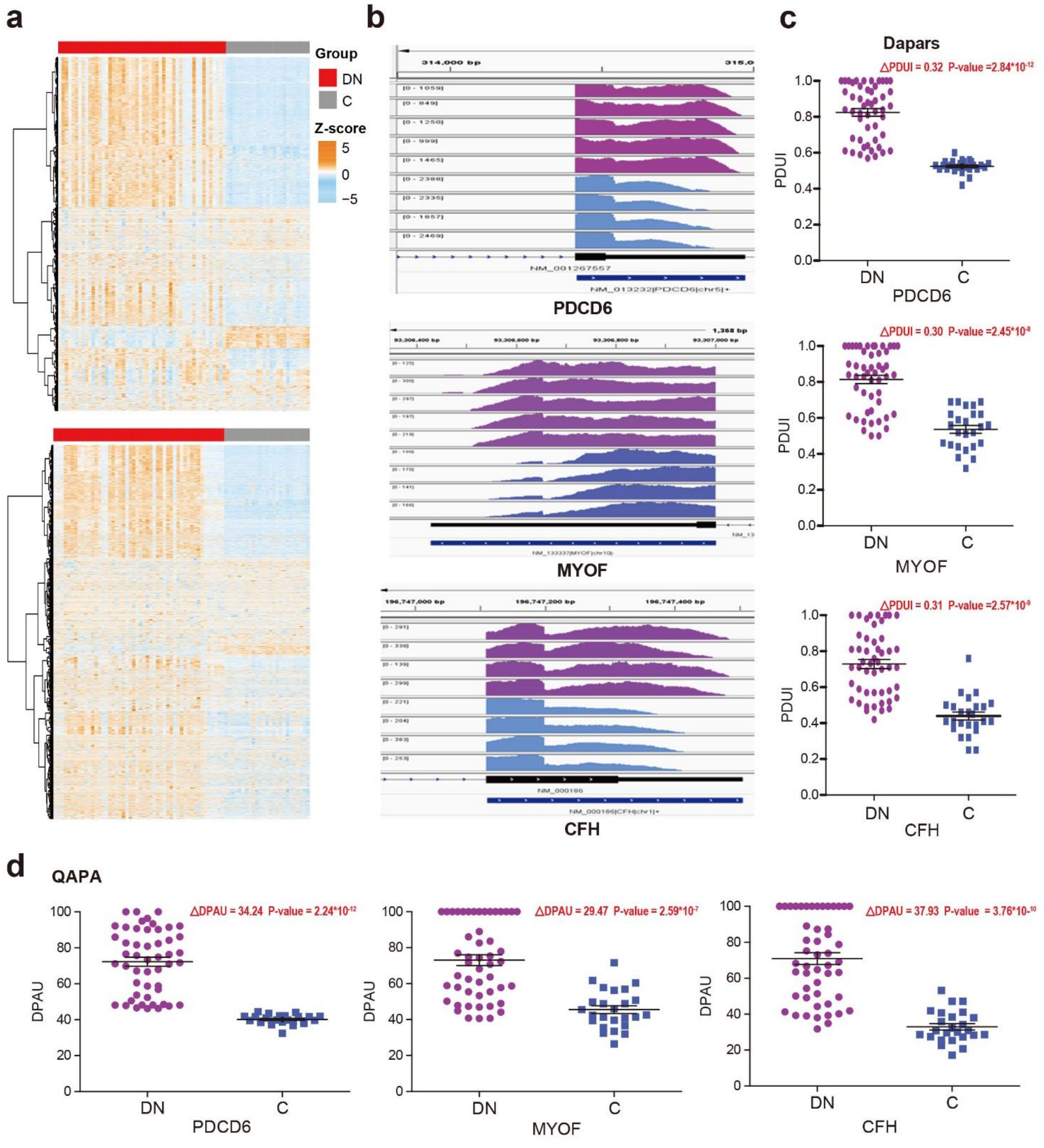

Figure S2. The global lengthening of 3′UTRs in DN and the representative examples of dynamic APA-regulated genes.

a, The heatmap of PDUI (top) and DPAU (bottom) depicted the global lengthening of 3′UTRs in DN patients compared to control subjects. Orange indicated 3′UTR lengthening genes, and blue indicated 3′UTR shortening genes. Each column represented a sample and each row represented a gene.

b, The visualization of RNA-seq tracks for the representative examples of dynamic APA-regulated genes (PDCD6, MYOF and CFH) between DN and control. The purple and blue tracks represented DN and control samples, respectively

c-d, The quantification of PDUI (c) and DPAU (d) changes for PDCD6, MYOF and CFH between DN and control samples.

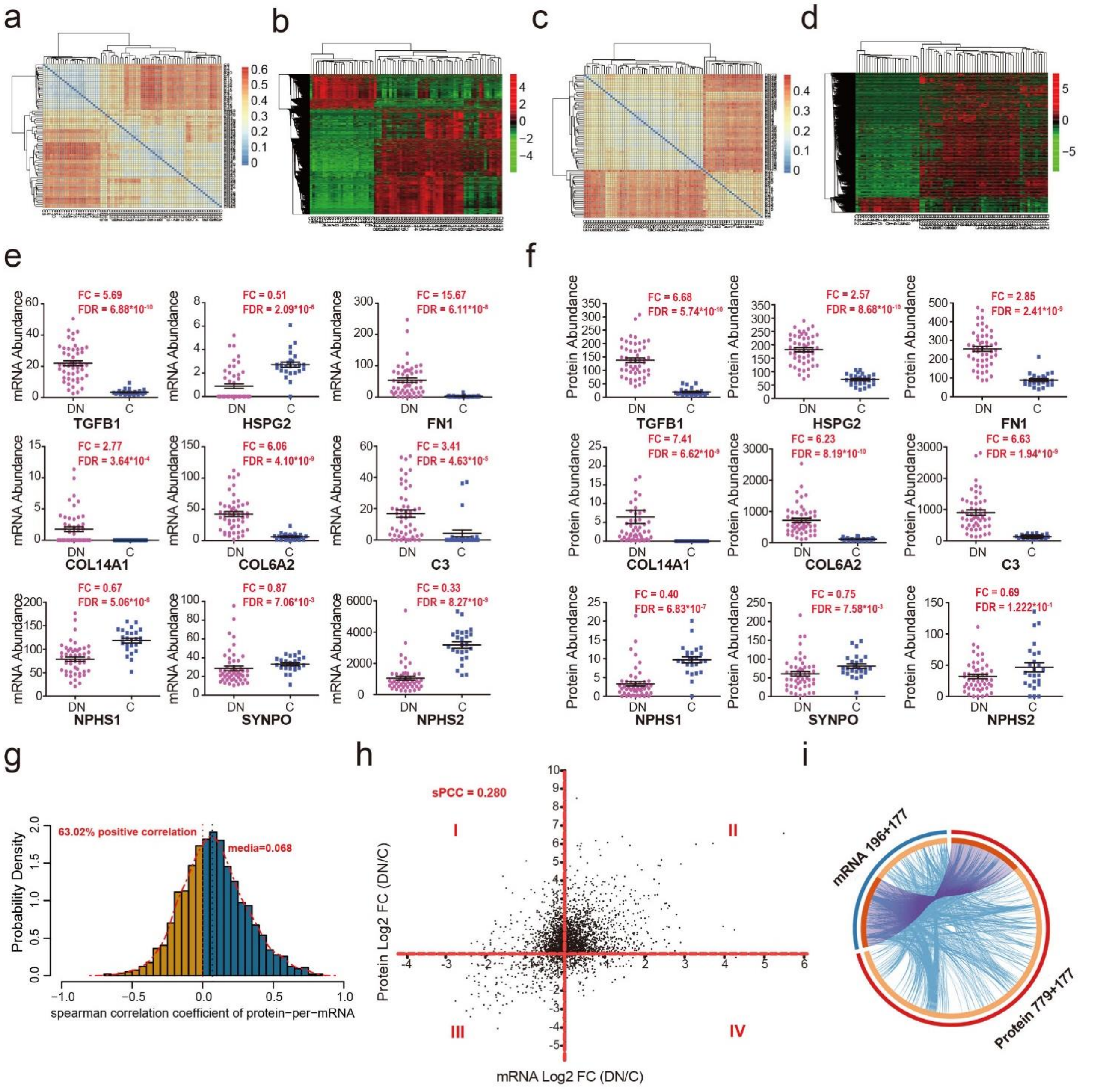

Figure S3. Transcriptomics and proteomics analysis of glomeruli isolated from DN patients and controls.

a, c, The unsupervised hierarchical clustering (HC) analysis based on the correlations of mRNA (a) and protein(c) abundance between samples. Each column and row represented a sample.

b, d, The heatmaps of the differentially expressed mRNAs (b) and proteins (d). Red indicated upregulated genes, and green indicated downregulated genes. Each column represented a sample, and each row represented a gene.

e, f, The mRNA (e) and proteins (f) scatterplots of the representative DN-associated genes (TGFB1, HSPG2, FN1 etc.) between DN patients and controls.

g, The distribution of protein-mRNA correlation of individual gene calculated by Spearman's correlation coefficient. The median Spearman's correlation coefficient r = 0.068 and 63.02% mRNA-protein pairs showed positive correlations (blue).

h, The correlation of log2FC (DN/C) between the mRNA and protein for the mRNA-protein matched genes. The Spearman's correlation coefficient r = 0.280. sPCC, Spearman's correlation coefficient.

i, The Circos plot showed how the differentially expressed mRNAs and proteins overlapped. On the outside, the blue arc represented mRNA list and the red arc represented protein list. On the middle, the dark and light orange arc represented the genes that appeared in both lists or uniquely to each list, respectively. On the inside, blue lines linked the genes where they fell into the same GO term and purple lines linked the same genes that were shared by both gene lists.

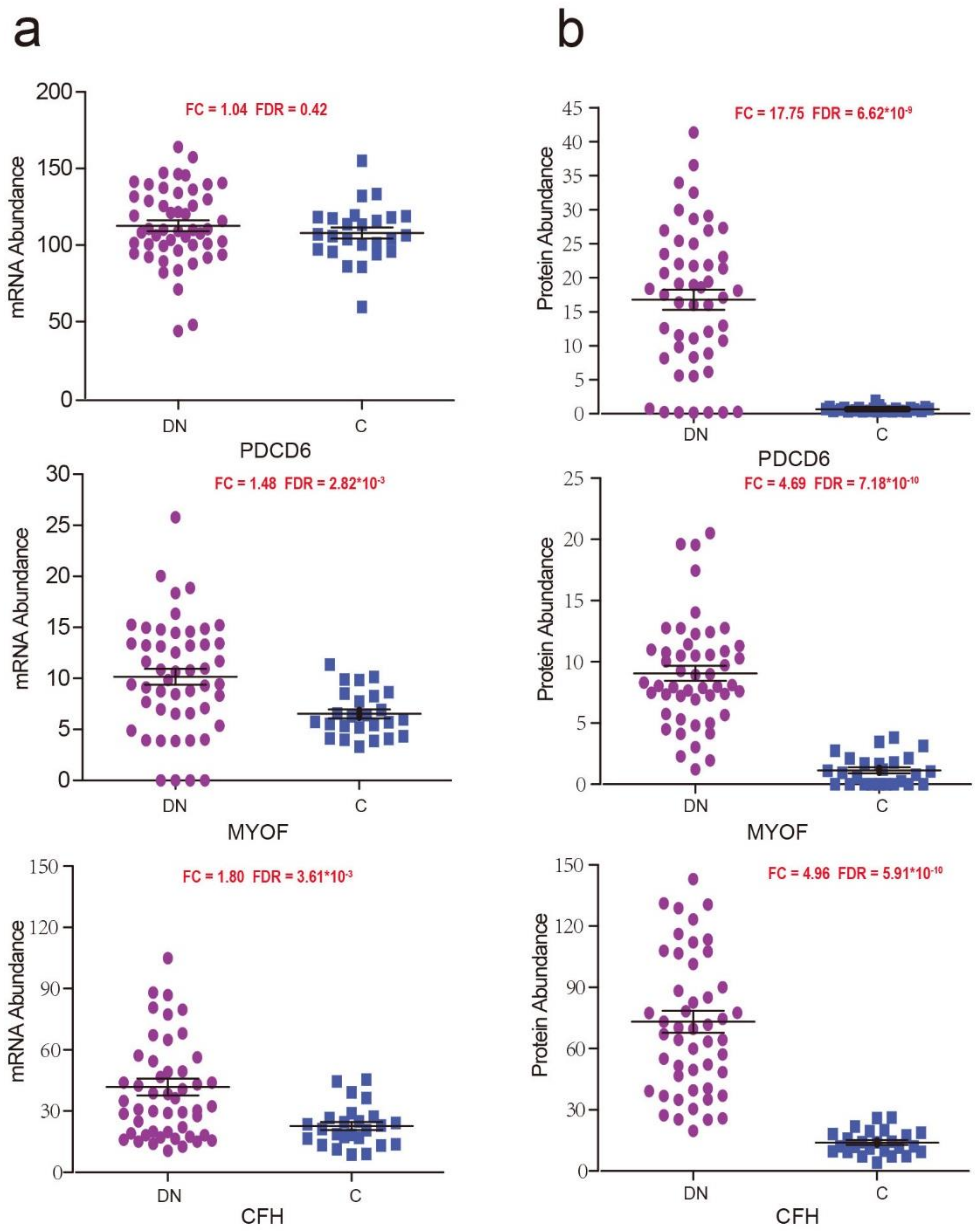

Figure S4. The mRNA and protein expression changes between DN and control for the represent APA-regulated genes.

a-b, The mRNA (a) and protein (b) scatterplots for the genes of PDCD6, MYOF and CFH between DN and control samples.

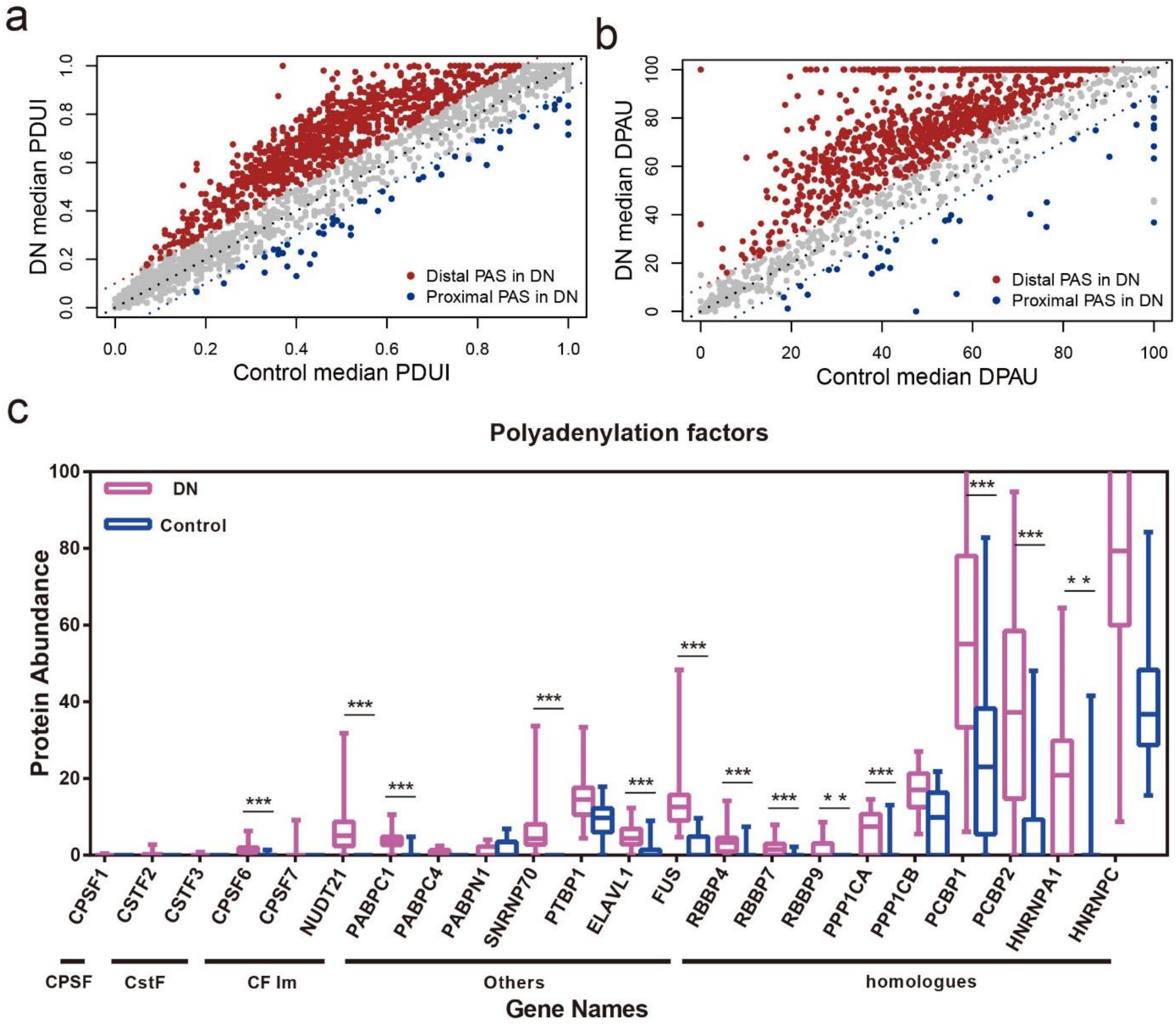

Figure S5. The global lengthening of 3′UTRs and the protein expression changes of the core polyadenylation factors in DN.

a-b, Scatterplots of median PDUI (a) and DPAU (b) score between DN and control samples for the 2,961 mRNA-protein matched genes.

c, The boxplot of protein expression changes for the known polyadenylation factors between DN and control samples based on proteomics data. The protein abundance for DN (red) and control (blue) samples were colored respectively. A factor was considered significantly differential expression if the FDR< 0.05 and the log2FC > 1. (***) FDR < 0.001, (**) FDR < 0.01 and (*) FDR < 0.05.

Legends for Supplementary Table S1 to S6

Table S1. The clinic characteristics for DN patients and controls.

Table S2. The primers for qRT-PCR and overexpression vector.

Table S3. The dynamic APA events calculated by Dapars and QAPA algorithms.

Table S4. The transcriptomics and proteomics data analysis.

Table S5. The protein-per-mRNA FC ratio analysis.

Table S6. The RBPs that significantly improved protein translation underlying DN.
